# Ontario family physicians’ perspectives about their scope of practice: what is it, what drives it and how does it change?

**DOI:** 10.1186/s12875-022-01833-5

**Published:** 2022-09-26

**Authors:** Sophia M. Myles, Elizabeth F. Wenghofer, Rachel H. Ellaway, Michael T. Yeo

**Affiliations:** 1 Institute of Health Policy, Management and Evaluation, University of Toronto, 155 College Street, Toronto, ON M5T 3M6 Canada; 2 School of Kinesiology and Health Sciences, Laurentian University, 935 Ramsey Lake Road, Sudbury, ON P3E 2C6 Canada; 3 Department of Community Health Sciences, Cumming School of Medicine, University of Calgary, 3330 Hospital Drive NW, Calgary, AB T2N 4N1 Canada; 4 Faculty of Arts, Laurentian University, 935 Ramsey Lake Road, Sudbury, ON P3E 2C6 Canada

**Keywords:** Scope of practice, Medicine, Family physicians, Physician perspective, Factors, Lived experience, Clinical and non-clinical practice

## Abstract

**Background:**

There is little evidence to show what scope of practice (SOP) means from the point of view of family physicians, how family physicians think about their SOP as it changes over time, or what factors shape and influence their SOP. Understanding family physician perspectives on SOP and the factors that influence it can aid our understanding of how it can constrain and enable physicians’ agency and autonomy in professional practice.

**Methods:**

Using qualitative description and incorporating constructivist grounded theory data collection and analysis techniques, four focus groups were conducted involving twenty-four Ontario-based family physicians from different contexts, at different career stages, and with different practice experiences.

**Results:**

Participants’ SOP was highly dynamic, changing throughout their careers due to factors both within and beyond their control. Their sense of their own SOP was the product of a continuous cycle of personal and professional transitions, exposures, and experiences throughout their careers. These family physicians sought regular and sustained mentorship, support, and engagement for their SOP throughout their careers. This was particularly the case during professional transitions and for drivers of their SOP for which they felt unprepared early in their careers, such as through the first years of independent practice, and when functioning as owner-operators of medical practices. Four descriptive themes were identified focusing on the nature of their current practice, their professional preparedness and supports, practice management dynamics, and ‘doctors are people, too’.

**Conclusions:**

The SOP of the family physicians in this study was dynamic and unique to each individual, it emerged from interactions between their personal and professional lives and identities, and it was embedded in their lived experiences. SOP was also to some extent imposed and externally driven. This study advances understanding by exploring the ‘why’ and ‘how’ of SOP rather than focusing solely on what it is.

## Background

Family physicians (FPs) are typically the coordinators and gate-keepers for their patients’ access to and navigation of health care systems in many developed and developing countries [1–6]. Although the responsibilities of FPs can encompass primary, secondary and tertiary levels of care [2], they mostly provide primary care services [7]. FPs also have the broadest potential patient base compared to other medical specialties, which requires a broad knowledge base which overlaps with that of many other medical specialties. Given the breadth of roles and responsibilities of FPs, it is important to understand the concept of scope of practice (SOP) of FPs in primary care—i.e., what these physicians actually do and what they are allowed to do in their professional practice throughout their careers.

Some researchers have explored primary care SOP using questionnaires and inventories, both in general [7–9] and in specific settings [10, 11]. Others have compared the intended and reported scopes of practice (SOPs) of family medicine residents [12, 13], explored residency program and individual practitioner characteristics [14], and compared SOP to family medicine program match rates [15]. Other SOP-related studies have focused on rural community practice [16], family physician career choices [17], and what administrative data can say about SOP [18]. At different times, FPs’ SOP has been discussed in terms of its breadth and rate of change [19, 20], its geographic variation [21–23], how it changes over time[24–28], how it encompasses special interests or focused practice areas [29, 30], and how it relates to burnout among new practitioners [31, 32]. The impact on FP SOP when they are working in primary care teams has also been explored [33, 34].

Perspectives on how FPs think about SOP are notably absent from this literature, creating a deficit in knowledge as to what factors influence their SOP or what control they have to direct it. Understanding FP perspectives on SOP can aid our understanding of the factors that shape FP practice and how they constrain and enable FPs’ agency in shaping their SOP. This study contributes to the growing literature on FP SOP by exploring practitioner perspectives. Our study addressed the question “how do FPs understand their own SOP, what factors influence their SOP, and how and why might these factors change their SOP throughout their careers?”.

## Methods

### Context

This study was conducted in the province of Ontario in Canada. Ontario has a population of 14.9 million people with a FP population of 16,990 encompassing family medicine (11,396), emergency family medicine (923), and general practice (4,671) representing approximately 36% of all FPs in Canada [35]. In 1993, general practice training in Canada was discontinued when the one-year rotating general practice internship was replaced with the family medicine residency program [36].

### Study approach

We used a qualitative description (QD) methodology [37] given our focus on description and QD’s strengths in investigating a poorly understood phenomenon and providing a robust description thereof [38–40]. QD study designs usually involve a combination of sampling, data collection and analysis stages [37]. Given that QD has no fixed methods for data collection or analysis, we used techniques from constructivist grounded theory (CGT) [41] for the data collection and analysis procedures [42] within this broader QD study design.

The techniques described below employed an iterative process to ensure that data collection and analysis occurred simultaneously throughout the research process such that earlier data collection informed subsequent data collection and analysis which remained open to new ideas. Moreover, the process by which codes and categories were devised during analysis reflected the data and considered many observations. Inductive line-by-line coding was used to identify broader groups of codes and themes, and variations and patterns in the data were sought by making comparisons – within or between cases, over time – throughout the analysis process. As we did not aspire to generate theory from this study, we employed three of the six characteristics commonly included in grounded theory study designs [43].

### Data collection and procedure: study population, sample size and strategy

We recruited FPs who (1) were licensed to practise medicine independently (i.e., not on a provisional, educational, or other practice license requiring supervision), (2) had a primary practice address in Ontario, and (3) were either FPs certified by the College of Family Physicians of Canada (CFPC), or general practitioners (GPs) neither certified by the CFPC nor certified by the Royal College of Physicians and Surgeons of Canada. We used purposive, convenience and snowball sampling to select potential participants from those attending continuing professional development (CPD) courses offered by the Ontario College of Family Physicians (OCFP) and family practice peer assessor meetings offered by the College of Physicians and Surgeons of Ontario (CPSO). We also sought to include CPSO FP and general practitioner assessor network leads and physicians among the Ontario FP population. Before the COVID-19 pandemic, physicians were invited to participate in focus groups in person or via teleconference. With the assistance of the OCFP and the CPSO, invitees were emailed a cover letter, an informed consent form containing pertinent information about the study, the research team as per COREQ guidelines [44], and a letter of support for the study from the CFPC.

Prior to each focus group, basic practice and demographic background information from participants was requested. The focus groups themselves, which lasted up to two hours, focused on five open-ended questions regarding participants’ understanding of their SOP and what factors determined, limited, shaped and changed their SOP (Table 1). The focus groups were audio recorded and transcribed verbatim using a transcription service. Minimal notes were taken during each discussion to ensure optimum facilitator engagement. However, memos and field notes [41] were made immediately after each focus group to document initial thoughts about the conversation. Participants were assigned a code corresponding with their focus group transcript, recording, notes and, if provided, practice and demographic information to ensure participant confidentiality.

**Table 1:** Focus group guide to explore family physicians’ perspectives of their own SOP

Set of Open-Ended Questions Asked During Each Focus Group
1) How do you understand your scope of practice? Please describe your practice/How would you describe your practice?
2) In your opinion, what drives or determines your scope of practice? What are important elements or factors that help shape your scope of practice? Limit your scope of practice?
3) Has your scope of practice changed throughout your career? If yes, how? To what factors do you attribute these changes in scope?
4) With regard to your scope of practice and practice environment, context or setting: (a) Did you know what you were getting into? (b) When did you realize the realities of your situation? (c) Under what conditions did this become apparent? (d) When or under what conditions you believe you should have been made aware of the realities of your practice?
5) Is there anything of importance I haven’t asked you regarding your scope of practice or that you wish to add?

The focus groups were conducted and facilitated by the principal investigator (SM) as part of her doctoral research. Her position as a non-physician trainee presented potential advantages and disadvantages in how she was perceived by participants and how they shaped their stories in this research. Perceptions about her role as a researcher may have encouraged participants to take various approaches to the information they shared – or did not share – about SOP in their practices. For example, participants may have felt that they could freely share their thoughts and experiences because they were speaking to someone who did not represent, work for, or have membership with organizations concerned with physician governance and practice (e.g., CPSO, CFPC). Alternatively, physicians may have been reserved with some information they shared with a non-colleague based on the perception that their statements might be misunderstood, misinterpreted, or feel judged by someone who is a member of the public outside the context of this research.

Participants were given an incentive of earning CPD credits if they successfully fulfilled the remainder of the CFPC’s reporting requirements for a linking learning to practice activity [45, 46] which would contribute towards their maintenance of certification and licensure.

### Data analysis

After a descriptive analysis of the basic practice and demographic background information was conducted by SM, all audio recordings and transcripts were listened to and read several times by SM and discussed with EW. In further discussions with EW, SM categorized the data, and additional notes and memos were made, added to, and compared with those made during data collection. Transcript content was continuously compared, analyzed, coded, and grouped on multiple levels [41] to combine the categories, concepts and meanings ascribed to them that emerged from this process [47]. During initial coding, data were studied line by line to devise categories and concepts. Initial codes were generated by labelling words, lines, or sections of the data to identify themes for further investigation. During focused coding, the most frequent initial codes were identified, sorted, combined, and organized [41]. Focused codes were generated into categories and incidents, and subsequently, further developed, refined [47] and grouped. The focused codes were analyzed during theoretical coding, the process which enables the saturation of core categories identified during focused coding, carefully considering how concepts and categories generated related to each other. Core categories represent themes in the analysis [48]. To validate and ensure representativeness of themes and sub-themes generated from the analysis, a member check via email was conducted during which participants were sent a copy of their focus group transcript and the constructed themes and subthemes attached to each statement. Participants could add, amend, clarify, or retract any of their statements. This member checking was intended to support the co-construction of knowledge between researchers and participants [41]. The research team worked collaboratively to further refine the analysis and presentation of results.

## Findings

Four focus groups of five to seven participants each were conducted involving a total of twenty-four Ontario-based FPs. Twenty-three of the twenty-four participants provided basic practice and demographic background information (Table 2). Reported focused practices or special interests included care of the elderly, mental health, sexual health, public health, and administrative medicine. Reported primary practice settings included solo and group office practice, and interdisciplinary team-based practice (e.g., family health team), while non-clinical settings included a student health service, a youth and social services organization, a coroner’s office, a retirement home, and Ontario’s medical regulatory authority. Reflections about the versatility of practice settings and the impact on SOP were reflected in the statements of participants who worked in different settings at different times of the year (e.g., locum coverage during summer months and in another setting for the remainder of the year), as well as in statements that identified how one’s practice changed over time regardless of whether their practice setting changed or stayed the same.

**Table 2:** Voluntary basic participant practice and demographic background information

Characteristic	N (%)
**Age**	*n* = 22 Mean = 48.2 (29–70)
**Gender**	
Male	11 (45.8)
Female	13 (54.2)
**Years in Practice**	*n* = 23 Mean = 19.9 (< 1–43)
**Country of Undergraduate Medical Education**	
Canada	18 (81.8)
United Kingdom	4 (18.2)
**Primary Practice Address**	
Southern Ontario	16 (80)
Northern Ontario	4 (20)
**Primary Practice Activities**	
Comprehensive Family Practice	17 (77.3)
Focused/Special Interest Practice	5 (22.7)
**Primary Practice Setting**	
Solo Office Practice	2 (9.5)
Clinical Group Office Practice	7 (33.3)
FHT or Other Interdisciplinary Team-Based Practice	5 (23.8)
Hospital	2 (9.5)
Other	5 (23.8)

**Fig. 1:**
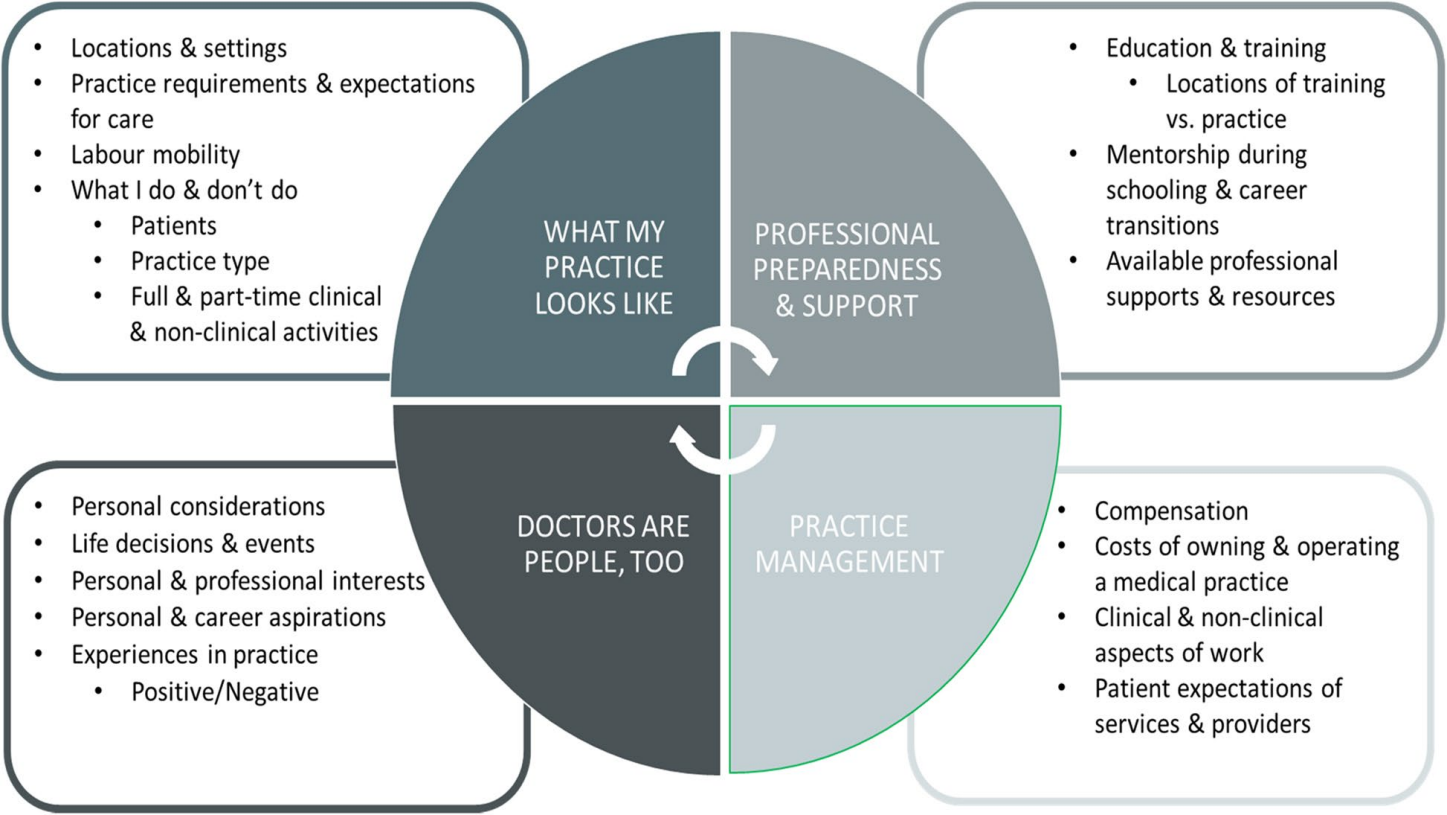
How Ontario Family Physicians Understand Their Own SOP

Participants’ SOP was the result of a continuous cycle of personal and professional transitions, exposures, and experiences throughout their careers. We grouped our findings around four main themes: (1) what my practice looks like; (2) professional preparedness and support; (3) practice management; and (4) doctors are people, too (Fig. 1).

### Theme 1: what my practice looks like

The locations and settings in which FPs practice were a major influence on their SOP. Practice requirements, expectations for care in different environments, and labour mobility were all described as impacting SOP. Participants also equated SOP with the patients they saw, the type of practice they had, their clinical and non-clinical practice activities, and the time they allocated to these activities.

Participants also saw geography and the practice environment as key elements in determining their SOP. The communities in which physicians practiced, the needs of those communities, and regional and jurisdictional variations in healthcare delivery also shaped SOP, *“I was practicing in an Aboriginal setting … I was just putting Band-Aids on very profound social problems. It really was my inspiration to go into public health”* (FG2-P10—male mid-career doctor in Southern Ontario). Further, expectations of colleagues and the standard of care expected in the community also shapes SOP:

*[My husband and I] wanted to do comprehensive family practice [and] be at a hospital that was family doctor run … once you’re in [a small, rural community], you are a necessary part of the functioning of that hospital and that community, and your practices – it’s a lot easier to pick up work than it is to step away from work because there’s an entire community and colleague network that relies on you …* (FG2-P8 - female mid-career doctor in Southern Ontario)

Some participants felt unprepared for the requirements of comprehensive practice, despite understanding that caring for rural and isolated populations required broad generalists able to undertake a broad SOP. FPs with experience practising in rural areas overwhelmingly reported having broader SOPs compared to their urban practising counterparts.

Participants also discussed the variability, versatility, diversity, and unique nature of family medicine and what it meant to their professional identity as FPs:

*Family practice is quite unique … in that you can go from [one area of practice to another]… [I do] locum work over the summer, and [my SOP will] be much, much broader; and then during the school [year] I do something that’s … more narrow. [This doesn’t change my being a family doctor]. It just simply changes my [SOP] …* (FG2-P8 – female mid-career doctor in Southern Ontario)

Participants described their SOP in terms of the procedures they performed, the services and types of care they provided, the kinds of environments in which they worked, and the institutional (credentialing committee) policies they were subject to. Their SOP was also shaped by the needs of ‘who walks in the door’ on any given day as well as a choice or interest to serve specific patient populations or groups. Several participants noted that their practice had grown (up) and aged with them, *“ … now I'm doing less pediatrics and well woman care, and more elder care”* (FG3-P19—male mid-career doctor in Northern Ontario). Participants also reported allocating time to specific areas of care according to the disease profile of their patients – e.g., treating chronic diseases linked to high rates of COPD.

Practice type or arrangement and time devoted to practice activities also had an impact on SOP. Some participants discussed how the SOP of a solo practitioner differed from a practitioner in a group practice. In addition to the areas of care for an FP’s own practice, responsibilities might also include cover as part of on-call services. Participants described both clinical and non-clinical tasks or practice commitments within their SOP, *“… 30 percent of my week is education focused in a teaching sphere, so I should probably not forget that … it's a massive part of my job”* (FG4-P22—female early career doctor in Southern Ontario).

### Theme 2: professional preparedness and support

This theme focused on what prepares and supports FPs in practice. We identified three factors that drove this aspect of FPs’ SOP: 1) their education, training, and the locations in which they trained and practice; 2) the mentorship they received during their training and subsequently, including career and practice transitions; and 3) their access to professional supports. We consider each in turn.

Several participants identified the influence of their education and training on their SOP. Exposure to different family practice environments during training allowed them to get a general sense of what different FP SOPs could involve. While some participants felt like these exposures had prepared them well, particularly if they ended up practising in places similar to where they trained, others felt that their training had not given them a full sense of the realities of family practice:

*Starting off as a locum in a practice you’re, I think as a resident, [really shielded] from real life of practicing medicine … I envisioned … I’d be doing a lot of different … things or more things that I’m currently doing … I’m working covering two locums at once … stuck at the office until eight or nine doing paperwork, so I did not expect it.* (FG1-P6 *–* female early career doctor in Southern Ontario)

This was particularly pronounced for international medical graduates who described having to negotiate additional challenges in understanding how FPs work in Canada.

Participants also discussed how the mentorship they had received during their education, both through CPD activities and throughout their professional practice, had informed and shaped their SOP. Some had sought mentorship and career advice on careers and variances in SOP, and their compatibility with life decisions:

*It would've been really nice before I sunk all this money into … extra training and [ courses and doing stuff abroad] to get that experience if someone had told me listen … [your spouse] has no desire to leave Canada, you should probably rethink [what you want to do] …* (FG4-P20 - male early career doctor in Southern Ontario)

Some participants suggested that CPD should have more of a focus on training, job opportunities, and financial and personal considerations to help them realistically assess their SOP and how it changed throughout their careers. Participants also reported experiencing a significant learning curve during the transition from residency training into independent practice and thought that robust mentorship during this critical juncture would have helped them orient to their SOP more effectively.

In addition to mentorship, participants identified the importance of aligning with the work of their clinical colleagues as a way of determining their own SOP, *“I gave up obstetrics … partly because there were four family doctors doing obstetrics [in the city in which I practise]”* (FG2-P12—female late career doctor in Southern Ontario). Where specialists were not available, care typically was offloaded to the primary care physician, *“I have done two appendectomies, a couple of hernias, tons of surgical assisting because we didn’t have other people to specialize and do this …”* (FG3-P17 – male late career doctor in Northern Ontario). Some participants reported a reluctance to retire or scale back their practice out of concern for leaving their patients doctorless. The opportunity to recruit and retain FP colleagues to a practice also informed FP decisions to adjust their SOP in response to service vacancies.

### Theme 3: practice management

This theme focused on the policies that shaped how FPs managed their practices, which in turn shaped their SOP. This included how and how much they got paid, what they did and did not get compensated for, the costs of owning and operating their practices, and clinical and non-clinical aspects of their work. Practice management issues also included the expectations of healthcare consumers regarding the services they received and the providers of those services.

Participants often attributed their SOP or changes in their SOP as reactions to how the health care system was financed and how FPs were remunerated. For example, several participants discussed being incentivized by being paid for certain services, while others described scenarios in which they had given up some aspect of practice they found enjoyable for something less enjoyable but for which they were better compensated. Areas of care not covered within provincial health insurance plans also limited what FPs incorporated within their SOP. For instance, several participants described offering travel medicine services but only to the extent that there was remuneration for those services.

Participants also identified their roles as business owners as part of their SOP. Costs associated with owning and operating a medical practice impacted what FPs decided to do or could afford to provide to their patients. The need to undertake administrative duties to offset the costs of running a practice also impacted the time FPs could devote to patient care. The business aspect of medicine was therefore a key aspect and driver of FP SOP:

*I don't think we're prepared for all of the administrative, non-medical stuff that goes along with medicine … 50 percent of [the] stuff that I do in a day that's not ‘medicine related’ including bureaucracies and policies of working for a large downtown hospital [was a] bit of a surprise to me.* (FG4-P22 – female early career doctor in Southern Ontario)

Although some participants acknowledged they had some training in practice management in residency training, there was a general sense of having been *“poorly trained”* or *“badly warned”* about these realities (FG4-P24—male late career doctor in Southern Ontario). It was suggested that the profession could do more to educate trainees about the non-medical side of practice to address some of their expectations for practice.

Additionally, the demands of healthcare consumers influence SOP, particularly in cases where patients demanded certain services that were not medically necessary and would have placed unnecessary financial costs on the healthcare system:

*There's just this very high expectation that … I ask for this, this is what I want, give it to me or I'm going to complain. And that is a very difficult thing to manage in a publicly funded [system], and I think that really drives our [SOP] as well … there are many days when I feel … that I've been more of a service provider than I have been … a physician. That I am managing … peoples' expectations [rather than] their illness.* (FG3-P16 - female mid-career doctor in Southern Ontario)

Participants also reflected on the impact of the Internet, increased patient access to information, and their desire to seek expertise outside the doctor’s office (e.g., ‘Dr. Google’) as contributing to shifting patient expectations and changes in doctor-patient interactions to more of a supplier–consumer relationship. Participants welcomed patient involvement in their care yet noted that this could change what constitutes medical expertise and who a medical expert is.

### Theme 4: doctors are people, too

This theme focused on personal considerations, life decisions and events, interests and aspirations, experiences in practice, and how the interactions of these factors impacted what physicians were able to do in practice throughout their careers.

Participants cautioned against making sweeping judgements about SOP because it was unique to each physician:

I think we have to be very cautious in making judgements from one physician to another as to the reasons that people have become more focused in their practice or remained broad based … because many of these things are also determined by family circumstances, relationships, partners, children … I've been fortunate … to do most of the things that I like for [much] of my career[. I’m not sure if that’s common]. (FG4-P24 – male late career doctor in Southern Ontario)

SOP was often characterized as reflecting the progression and intersection of personal, family, and professional lives. For instance, some participants discussed aspects of practice they would have liked to have incorporated in their SOP but that were no longer feasible, *“I had full intention of doing obstetrics … but had another baby myself and thought … I really couldn’t cope with that … it [was something] I needed to give up at that point”* (FG2-P13—female mid-career doctor in Southern Ontario). Work-life balance was a factor for both male and female FPs in shaping their SOP. For instance, several participants discussed the support they had needed from their families to maintain some aspects of their SOP (e.g., helping them shovel the driveway in the middle of the night to deliver a baby). Spousal happiness and employment factors, particularly in physician-physician partnerships, also influenced where participants practiced, which also shaped their SOP:

*My husband is a physician as well, and we have a family… [My SOP has largely resulted from] our personal needs …I gave up [emergency medicine] because one of our children got sick. When something else happened in the family, my [SOP] had to change again … We are [parents and care givers] for our famil[ies]. Over time, it has had a massive influence on where I practice and why I practice the way I do.* (FG2-P8 - female mid-career doctor in Southern Ontario)

Participants indicated that their priorities, practice intentions and resulting SOP were not static. For instance, SOPs often shrunk or expanded depending on the age of children, and practices would be scaled back to spend more time with grandchildren.

Age and career stage impacted both anticipated and actual SOPs, *“when I finished my training … [I thought I was going to have a bigger SOP] … I’m still early [in my career] so I still have lots of time to do (other) things”* (FG1-P6—female early career doctor in Southern Ontario). Several mid- and late-career participants discussed how their SOP had evolved as their careers had changed over time:

*My [SOP] has changed over my career. It is in many places a matter of emphasis. When I started my career, there was a heavy emphasis on emergency and internal medicine on the acute care ward. [As] time went on, the emphasis moved more towards the clinic.* (FG3-P19 - male mid-career doctor in Northern Ontario)

Several participants mentioned incorporating non-clinical roles or jobs at different stages in their careers, such as administrative management and regulatory college assessments. Regardless of age and career stage, many participants discussed personal health issues that had limited their SOP and work hours, such as withdrawing into 9 to 5 practice. Feelings of burnout were a recurring issue, *“… I became weary of working 80 h a week, being in the office every weekend, rounding every day”* (FG2-P9—male late career doctor in Southern Ontario). Such demanding schedules made it difficult for some participants to achieve a work-life balance.

Personal and professional interests also influenced FP SOP. Participants reported their SOP had been shaped by areas of practice they were interested in (e.g., sports medicine and palliative care) and by special-interest issues (e.g., homelessness and inner-city medicine). Negative experiences also shaped SOP, *“My practice has been … [shaped by] bad experience to some degree[.] I did obstetrics for a while [and] I found it very difficult”* (FG1-P1—female late career doctor in Southern Ontario). What FPs were comfortable doing, what opportunities they had, and what they considered to be a strength of their practice also changed over time, *“I went into occupational medicine [because] one of my patients was a physician … when he was retiring, he said ‘would you like to take over my job?’…”* (FG3-P18—male late career doctor in Northern Ontario). While most participants had been intrigued by professional opportunities afforded them at different stages in their careers, job stability and sustainability often determined what aspects of SOP were feasible or realistic to pursue or maintain over the long term.

## Discussion

SOP is primarily understood as reflecting the breadth of services and kinds of care that a physician can or does provide. Participants in this study understood their SOP and the factors that impacted their SOP over time in terms of what their practice looked like, their professional preparedness and supports, practice management issues, and who they were as people and in their lives outside of work. Some of the factors identified related to the practice environment and education and training are similar to those previously identified in the SOP literature [7, 8, 14, 16, 17, 21, 22]. However, the themes related to the business and personal aspects of being a doctor have not been well documented in this literature which may partly be due to assumptions that SOP is driven primarily by factors that are exclusively at the level of direct physician–patient clinical interactions. Our findings suggest that SOP encompasses much more than clinical family practice. They also suggest a need to rethink the artificial separation between physician and practice factors to more accurately reflect the mutual influence among personal and professional interactions that comprise FPs’ lived experiences.

A key finding from this study was that, regardless of age, career stage, gender, or practice context, FPs are people, too, and that has a significant impact on their SOP. For instance, family life was cited as a major consideration for how, where, when, or even if, a FP will practice. Broadly, our findings are consistent with factors and considerations in the recruitment and retention literature and practice intention literature that has identified practice support [12, 49]; employer constraints [12]; exposure to training in similar settings/environments [50, 51]; supportive work environment [50]; family and spousal support including partner employment considerations [50, 52, 53]; life plans, burnout [52, 53]; work-life balance, and remuneration [49, 51, 54, 55]. Our findings also included or touched on factors similar to those identified by studies on satisfaction of trainees and GPs in primary care in the European context. These themes include GPs as people, the practitioner-patient relationship, workplace autonomy, work-life balance including private life, relatives, and family [56, 57]; commitment to general practice, skills and competencies for general practice [57]; balance between workload and income, professional challenges or responsibilities such as teaching [58].

Participants also identified non-clinical aspects of their practice (i.e., activities outside of direct patient care) as being part of their SOP. For example, many participants indicated that they were ill-prepared to function as owner-operators of medical practices early in their careers. Globally, physicians balance dual identities of health care provider and small business owner [59–64] in publicly funded health systems. Certification bodies and CPD providers might consider this aspect of SOP when structuring educational and practice support for physicians.

Reitz and colleagues [17] identified factors that had contributed to the decisions of FPs in the United States to pursue and continue full-spectrum rural medical practice. Their participants *“describe[d] large shifts in their scope across a career, but took satisfaction in their [discretion] to make these changes at their pace and level of readiness”* [17]. Our findings indicate that FPs’ SOP changes over time due to numerous factors both within and beyond their control. Some participants felt they had considerable agency to make SOP changes as they wished, while others felt their discretion was significantly limited due to personal and professional circumstances. While dissatisfaction could potentially lead to a physician changing their SOP to be more satisfying (which may also involve a change in practice location or type), collectively, these findings raise questions about the perceived level of discretion and the actual level of control FPs possess over their SOP.

There were limitations to this study. There was potential for social acceptability bias in participant responses. To mitigate this bias, we maintained confidentiality by assigning unique participant codes corresponding with identifying materials. There was also potential for researcher bias with the participant selection and sampling strategies used to counteract non-response, low response, and participation rates of physicians in research [65–68]. While targeting specific groups of physicians contributed to a small number of focus groups, theoretical saturation was reached [41]. Participant statements predominantly addressed different aspects of the four themes such that no additional themes could be generated. Researcher bias may also impact how participant responses were interpreted. To counteract these biases, research team deliberations, member-checking and rigorous analysis procedures were used. Additionally, the focus on family medicine in Ontario could limit the generalizability of our findings. However, differences within other subnational jurisdictions and countries are equally complex, and the considerations identified within family medicine are likely similar to those experienced in other medical specialties and professions. Thus, while specialty, health professional and geographic specifics may vary, we believe these findings to be generally applicable.

Despite these limitations, this study encouraged the generation of a practice and evidence-informed understanding of SOP, emphasizing physician voices. FPs seek regular and sustained engagement, support, and mentorship throughout their careers for drivers of their SOP for which they feel unprepared and during professional transitions, particularly from residency through the first several years in independent practice. This study also shows that FPs adapt their practice to their community, to the providers around them, and to the care needs of the population and of their patients; in the same context, FPs can have different SOPs. Additionally, this study highlights that SOP is personal to each physician, balancing personal and professional considerations when deciding what to do in practice throughout their careers. As SOP evolves throughout FP careers, we need to view FPs wholistically as people rather than exclusively as professionals. This tension can result in potential gaps in agency of what FPs would prefer to do, are able to do, what opportunities are available to them and where, and what is required of them to meet community or population needs. FPs may be more or less prepared by their training for future SOPs, particularly where they must compromise their hoped-for SOP to fit the realities of the positions they find themselves in. The link between training preparing early career physicians for their SOPs could be the focus of a subsequent study.

Future investigations could address the expectations patients or colleagues have of physicians for their SOP. Future studies could also examine the gap between physician SOP, the SOP required of physicians to meet population needs and its implications on equitable care and patient safety. Future research could also explore changes in physician SOP because of COVID-19 [69], the widespread shift to virtual care [70], and required supports for these changes considering the factors identified in this study.

## Conclusions

Our analysis of FP perspectives on SOP, informed by their experiences with their own SOPs, represents a significant contribution to the literature, not least by addressing previous lacunae in what issues might impact SOP. Despite being situated in different contexts, at different career stages, and having different practice experiences, these physicians understood their SOP in terms of what their practice looked like, their professional preparedness and supports, their practice management responsibilities, and who they were as people and their life outside of work. SOP is unique to each physician, emerges from interactions between their personal and professional lives, and is embedded in their lived experiences. A main driver of SOP throughout FP careers was work-life balance. For many FPs, their SOP changed and evolved due to factors within and beyond their control. This raises broader questions about the perceived and realized agency FPs have over their SOP, which encompasses so much more than day-to-day clinical practice.

## Data Availability

The datasets generated and/or used during the current study are not publicly available due to ethical restrictions. Participants of this study did not agree for their data to be shared publicly. The corresponding author can be contacted for more information, however individual-level data have not been approved for sharing.

## Abbreviations

FPs: Family physicians
SOP: Scope of Practice
SOPs: Scopes of Practice
FP: Family physician
CFPC: College of Family Physicians of Canada
QD: Qualitative Description
CPD: Continuing professional development
OCFP: Ontario College of Family Physicians
CPSO: College of Physicians and Surgeons of Ontario
COPD: Chronic Obstructive Pulmonary Disease

## References

[1] Gross R, Tabenkin H, Brammli-Greenberg S (2000). Who needs a gatekeeper? Patients' views of the role of the primary care physician. Fam Pract.

[2] Flood CM, Downie J, Caulfield T, Flood C (2002). The Anatomy of Medicare. Canadian Health Law and Policy.

[3] Schwenkglenks M, Preiswerk G, Lehner R, Weber F, Szucs TD (2006). Economic efficiency of gate-keeping compared with fee for service plans: a Swiss example. J Epidemiol Community Health.

[4] van Loenen T, van den Berg MJ, Heinemann S, Baker R, Faber MJ, Westert GP (2016). Trends towards stronger primary care in three western European countries; 2006–2012. BMC Fam Pract.

[5] Gan Y, Li W, Cao S (2016). Patients' willingness on community health centers as gatekeepers and associated factors in Shenzhen, China: a cross-sectional study. Medicine (Baltimore).

[6] Liang C, Mei J, Liang Y, Hu R, Li L, Kuang L (2019). The effects of gatekeeping on the quality of primary care in Guangdong Province, China: a cross-sectional study using primary care assessment tool-adult edition. BMC Fam Pract.

[7] Wong E, Stewart M (2010). Predicting the scope of practice of family physicians. Can Fam Physician.

[8] Ie K, Ichikawa S, Takemura YC (2015). Development of a questionnaire to measure primary care physicians’ scope of practice. BMC Fam Pract.

[9] O'Neill T, Peabody MR, Blackburn BE, Peterson LE (2014). Creating the Individual Scope of Practice (I-SOP) scale. J Appl Meas.

[10] Baker E, Schmitz D, Epperly T, Nukui A, Miller CM (2010). Rural Idaho family physicians' scope of practice. J Rural Health.

[11] Hutten-Czapski P, Pitblado R, Slade S (2004). Short report: Scope of family practice in rural and urban settings. Can Fam Physician.

[12] Coutinho AJ, Cochrane A, Stelter K, Phillips RL, Peterson LE (2015). Comparison of intended scope of practice for family medicine residents with reported scope of practice among practicing family physicians. JAMA.

[13] Barreto TW, Eden AR, Petterson S, Bazemore AW, Peterson LE (2017). Intention versus reality: Family medicine residency graduates' intention to practice obstetrics. J Am Board Fam Med.

[14] Coutinho AJ, Levin Z, Petterson S, Phillips RL, Peterson LE (2019). Residency program characteristics and individual physician practice characteristics associated with family physician scope of practice. Acad Med.

[15] Anthony D, White J, Margo K, Tarn DM (2017). Scope of practice and family medicine match rates: results from a CERA clerkship directors' survey. Fam Med.

[16] Skariah JM, Rasmussen C, Hollander-Rodriguez J (2017). Rural curricular guidelines based on practice scope of recent residency graduates practicing in small communities. Fam Med.

[17] Reitz R, Horst K, Davenport M, Klemmetsen S, Clark M (2018). Factors influencing family physician scope of practice: a grounded theory study. Fam Med.

[18] Katz A, Halas G, Dillon M (2012). Describing the content of primary care: limitations of Canadian billing data. BMC Fam Pract.

[19] Beaulieu MD, Rioux M, Rocher G, Samson L, Boucher L (2008). Family practice: professional identity in transition. A case study of family medicine in Canada. Soc Sci Med.

[20] Ringdahl E, Delzell JE, Kruse RL (2006). Changing practice patterns of family medicine graduates: a comparison of alumni surveys from 1998 to 2004. J Am Board Fam Med.

[21] Myhre D, Szafran O, Schipper S, Dickinson J, Janke F (2018). Scope of practice of family medicine graduates who completed a rural versus urban program. Rural Remote Health.

[22] Wenghofer EF, Kam SM, Timony PE, Strasser RP, Sutinen J (2018). Geographic variation in family physician and general practitioner scope of practice in Ontario: a comparative provincial study. Can Fam Physician.

[23] Nasim U, Morgan ZJ, Peterson LE. The Declining Scope of Practice of Family Physicians Is Limited to Urban Areas. J Rural Health. 2020. 10.1111/jrh.12540 10.1111/jrh.12540 33244807

[24] Carek PJ (2018). Potentially alarming trends in the scope of practice for family physicians. J Am Board Fam Med.

[25] Peterson LE, Fang B, Puffer JC, Bazemore AW (2018). Wide gap between preparation and scope of practice of early career family physicians. J Am Board Fam Med.

[26] Weidner AKH, Chen FM (2019). Changes in preparation and practice patterns among new family physicians. Ann Fam Med.

[27] Carek PJ (2019). Declining presence of family physicians in hospital-based care: A major concern or totally makes sense?. J Am Board Fam Med.

[28] Jetty A, Jabbarpour Y, Petterson S, Eden A, Bazemore A (2019). The declining presence of family physicians in hospital-based care. J Am Board Fam Med.

[29] Gutkin C. Special interest areas in your practice?: Tell us more. Can Fam Physician. 2011;57(9):1096, 1095, 1094–5. PMID: 21918155. PMC3173436 21918155

[30] Sisler JJ, DeCarolis M, Robinson D, Sivananthan G (2013). Family physicians who have focused practices in oncology: results of a national survey. Can Fam Physician.

[31] Eiff MP, Hollander-Rodriguez J, Skariah J, Young R, Waller E, Dexter E, O'Neill TR, Peabody MR, Green LA, Carney PA (2017). Scope of practice among recent family medicine residency graduates. Fam Med.

[32] Weidner AKH, Phillips RL, Fang B, Peterson LE (2018). Burnout and scope of practice in new family physicians. Ann Fam Med.

[33] Dai M, Ingham RC, Peterson LE (2019). Scope of practice and patient panel size of family physicians who work with nurse practitioners or physician assistants. Fam Med.

[34] Freeman J (2019). Family physicians, nurse practitioners, physician assistants, and scope of practice: who will decide?. Fam Med.

[35] Canadian Institute for Health Information (2021). Supply, Distribution and Migration of Physicians in Canada, 2020 — Data Tables.

[36] Evans RG, McGrail KM (2008). Richard III, Barer-Stoddart and the Daughter of Time. Healthc Policy.

[37] Sandelowski M (2000). Whatever happened to qualitative description?. Res Nurs Health.

[38] Kim H, Sefcik JS, Bradway C (2017). Characteristics of qualitative descriptive studies: a systematic review. Res Nurs Health.

[39] Neergaard MA, Olesen F, Andersen RS, Sondergaard J (2009). Qualitative description - the poor cousin of health research?. BMC Med Res Methodol.

[40] Sullivan-Bolyai S, Bova C, Harper D (2005). Developing and refining interventions in persons with health disparities: The use of qualitative description. Nurs Outlook.

[41] Charmaz K (2006). Constructing Grounded Theory: A Practical Guide Through Qualitative Analysis.

[42] Kennedy TJ, Lingard LA (2006). Making sense of grounded theory in medical education. Med Educ.

[43] Hutchison AJ, Johnston L, Breckon J (2011). Grounded theory-based research within exercise psychology: a critical review. Qual Res Psychol.

[44] Tong A, Sainsbury P, Craig J (2007). Consolidated criteria for reporting qualitative research (COREQ): a 32-item checklist for interviews and focus groups. Int J Qual Health Care.

[45] College of Family Physicians of Canada. Linking learning exercises. Mississauga, ON: CFPC; 2020 [cited 1 July 2021]. Available from: https://www.cfpc.ca/en/education-professional-development/cpd-at-cfpc/linking-learning-exercises.

[46] College of Family Physicians of Canada. n.d. Linking earning to practice submission form for Mainpro-C credits. Mississauga, ON: CFPC; n.d. [cited 1 July 2021]. Available from: http://www.med.uottawa.ca/cme/assets/documents/Linking_Learning_to_Practice.pdf.

[47] Holtslander L (2007). Searching for new hope: A grounded theory of the experience of hope for older women who are bereaved palliative caregivers [dissertation].

[48] Alemu G, Stevens B, Ross P, Chandler J (2017). The use of a constructivist grounded theory method to explore the role of socially-constructed metadata (Web 2.0) approaches. QQML.

[49] Duffrin C, Cashion M, Cummings DM, Whetstone L, Firnhaber J, Levine G, Watson R, Lambert A (2016). Generational differences in practice site selection criteria amongst primary care physicians. Marshall Jo Med.

[50] Asghari S, Aubrey-Bassler K, Godwin M, Rourke J, Mathews M, Barnes P (2017). Factors influencing choice to practise in rural and remote communities throughout a physician’s career cycle. Can J Rural Med.

[51] Eide DB (2015). Key factors for physician recruitment and retention in rural hospitals [dissertation].

[52] Jolicoeur J, DeMiglio L, Kin LN, Orrantia E (2022). Why they leave: small town rural realities of northern physician turnover. Can J Rural Med.

[53] Mayo E, Mathews M (2006). Spousal perspectives on factors influencing recruitment and retention of rural family physicians. Can J Rural Med.

[54] Mathews M, Seguin M, Chowdhury N, Card RT (2012). A qualitative study of factors influencing different generations of Newfoundland and Saskatchewan trained physicians to leave a work location. Hum Resour Health.

[55] Parlier AB, Galvin SL, Thach S, Kruidenier D, Fagan EB (2018). The road to rural primary care: a narrative review of factors that help develop, recruit, and retain rural primary Care physicians. Acad Med.

[56] Le Floch B, Bastiaens H, Le Reste JY, Lingner H, Hoffman R, Czachowski S, Assenova R, Koskela TH, Klemenc-Ketis Z, Nabbe P, Sowinska A, Montier T, Peremans L (2019). Which positive factors give general practitioners job satisfaction and make general practice a rewarding career? A European multicentric qualitative research by the European general practice research network. BMC Fam Pract.

[57] Le Floch B, Bastiaens H, Le Reste JY, Nabbe P, Montier T, Peremans L (2022). Examining positive views from students, trainees and GPs about general practice: a generational problem? A set of qualitative studies in France. BMJ Open.

[58] Le Floch B, Bastiaens H, Le Reste JY, Lingner H, Hoffman RD, Czachowski S, Assenova R, Koskela TH, Klemenc-Ketis Z, Nabbe P, Sowinska A, Montier T, Peremans L (2016). Which positive factors determine the GP satisfaction in clinical practice? A systematic literature review. BMC Fam Pract.

[59] Calnan M, Collyer F (2015). Eliot Freidson: Sociological narratives of professionalism and modern medicine. The Palgrave Handbook of Social Theory in Health, Illness and Medicine.

[60] Spyridonidis D, Calnan M (2011). Are new forms of professionalism Emerging in medicine? The case of the implementation of NICE guidelines. Health Sociol Rev.

[61] Huby G, Guthrie B, Grant S, Watkins F, Checkland K, McDonald R (2008). Whither British general practice after the 2004 GMS contract?: Stories and realities of change in four UK general practices. JHOM.

[62] McDonald R, Harrison S, Checkland K (2008). Identity, contract and enterprise in a primary care setting: An English general practice case study. Organization.

[63] Chevreul K, Berg Brigham K, Durand-Zaleski I, Hernández-Quevedo C (2015). France: Health system review. Health Syst Transit.

[64] Goroll AH, Greiner AC, Schoenbaum SC (2021). Reform of payment for primary care—from evolution to revolution. NEJM.

[65] Cook JV, Dickinson HO, Eccles MP (2009). Response rates in postal surveys of healthcare professionals between 1996 and 2005: an Observational Study. BMC Health Serv Res.

[66] Thorpe C, Ryan B, McLean SL, Burt A, Stewart M, Brown JB (2009). How to obtain excellent response rates when surveying physicians. Fam Pract.

[67] VanGeest JB, Johnson TP, Welch VL (2007). Methodologies for improving response rates in surveys of physicians: a systematic review. Eval Health Prof.

[68] Wiebe ER, Kaczorowski J, MacKay J (2012). Why are response rates in clinician surveys declining?. Can Fam Physician.

[69] Kiran T, Green ME, Wu FC, Kopp A, Latifovic L, Frymire E, Glazier RH. Did the COVID-19 pandemic result in more family physicians stopping practice? Results from Ontario, Canada. medRxiv. 2021. 10.1101/2021.09.21.21263891

[70] Glazier RH, Green ME, Wu FC, Frymire E, Kopp A, Kiran T (2021). Shifts in office and virtual primary care during the early COVID-19 pandemic in Ontario. Canada CMAJ.

